# Fluorescence-Guided Surgery in Colorectal Cancer: State-of-the-Art and Translational Perspectives

**DOI:** 10.3390/curroncol33030160

**Published:** 2026-03-11

**Authors:** Florin-Alexandru Ruse, Dumitru-Cristinel Badiu, Cristian-Gabriel Popescu, Andreea-Ramona Treteanu, Anca Zgura, Octavian Andronic

**Affiliations:** 1 Faculty of Medicine, Carol Davila University of Medicine and Pharmacy, 050474 Bucharest, Romania; florin-alexandru.ruse0721@stud.umfcd.ro (F.-A.R.); andreea-ramona.treteanu0720@stud.umfcd.ro (A.-R.T.); 2 Department of General Surgery, Carol Davila University of Medicine and Pharmacy, 050474 Bucharest, Romania; cristianpopes@yahoo.com (C.-G.P.); octavian.andronic@umfcd.ro (O.A.); 3 Department of Oncology-Radiotherapy, Prof. Dr. Alexandru Trestioreanu Institute of Oncology, 022328 Bucuresti, Romania; anca.zgura@umfcd.ro; 4 Innovation and eHealth Center, Carol Davila University of Medicine and Pharmacy, 050474 Bucharest, Romania

**Keywords:** colorectal cancer, fluorescence-guided surgery, near-infrared, indocyanine green, anastomotic perfusion, lymphatic mapping, tumor localization, metastases

## Abstract

Background: Fluorescence-guided surgery based on near-infrared imaging, most often using indocyanine green (ICG), is increasingly used in colorectal cancer (CRC) surgery. This narrative review integrates current evidence across four clinically relevant domains-anastomotic perfusion, lymphatic mapping, tumor localization, and metastasis detection and emphasizes the technical and translational factors that will determine broader implementation. Methods: We performed a structured narrative review of clinical and translational studies identified through PubMed and citation tracking, with emphasis on ICG-based workflows and emerging targeted tracers. Because the literature spans heterogeneous interventions, imaging platforms, and endpoints, no de novo meta-analysis or formal risk-of-bias assessment was undertaken. Results: ICG fluorescence angiography is the most mature application and can refine transection-line selection, although its effect on anastomotic leak appears protocol dependent. In lymphatic mapping, ICG improves visualization of drainage pathways and nodal basins but does not reliably distinguish benign from metastatic nodes. For tumor localization, ICG supports lesion marking and dynamic tissue characterization, while targeted probes and contrast-free adjuncts may improve oncologic specificity. For metastatic disease, ICG is most useful for liver margin guidance and for excluding residual disease, whereas CEA-targeted and multimodal approaches appear particularly promising for peritoneal metastases. Conclusions: The added value of this review lies in linking current clinical maturity to the translational steps still required for routine adoption. In CRC surgery, fluorescence imaging is already useful in selected settings, but broader implementation will depend on standardized protocols, objective real-time quantification, and multicenter validation of targeted tracers against clinically meaningful outcomes.

## Simple Summary

Fluorescence-guided surgery is increasingly used in colorectal cancer to improve real-time intraoperative visualization. Near-infrared imaging with indocyanine green is already applied to assess anastomotic perfusion and map lymphatic drainage, while newer tumor-targeted tracers are being developed for lesion identification, margin assessment, and detection of peritoneal and hepatic metastases. This review summarizes current evidence across these applications and highlights the main barriers to wider adoption, particularly protocol standardization, objective fluorescence quantification, and validation of targeted probes.

## 1. Introduction

Colorectal cancer (CRC) ranks third worldwide in incidence and second in cancer-related mortality [1]. In operable stages, curative-intent resection remains the cornerstone of treatment, and intraoperative precision—particularly the accurate assessment of oncologic margins, perfusion of the resected segment, and occult or subtle disease extension—is decisive for both oncologic and functional outcomes. In practice, conventional visualization and palpation are often insufficient, especially in minimally invasive approaches, which has motivated the adoption of real-time optical techniques designed to complement standard perception and support surgical decision-making [2].

Fluorescence-guided surgery (FGS) addresses this need through the administration of a fluorophore and detection of its emission in the near-infrared (NIR) spectrum. Briefly, the molecule absorbs photons (excitation), transitions to an excited state, and subsequently returns to the ground state by emitting photons at a longer wavelength corresponding to lower photon energy—a phenomenon known as the Stokes shift. The ≈700–900 nm range is favorable intraoperatively, as tissues exhibit lower background autofluorescence and absorption than in the visible domain, resulting in a superior signal-to-noise ratio and increased effective penetration depth, thereby improving visualization of anatomical structures and perfusion dynamics [3]. Practical implementation relies on NIR cameras equipped with dedicated excitation sources and filters that overlay the fluorescent signal onto the white-light image on laparoscopic and robotic platforms, with similar options available for open surgery. Thus, FGS functionally extends the surgeon’s vision and reduces intraoperative uncertainty regarding the transection line, tissue viability, and subtle lesions [2,3,4].

Among the available fluorophores, indocyanine green (ICG) represents the current standard. It has an absorption peak around 750–800 nm, emission near 830 nm, high plasma protein binding, and an initial intravascular distribution that is favorable for fluorescence angiography. In colorectal surgery, ICG is predominantly used for assessing anastomotic perfusion and segmental intestinal vascularization; additionally, it serves for tumor marking (via submucosal or subserosal peritumoral injection) and, in certain protocols, for lymphatic mapping [4]. Recent literature suggests that ICG may guide transection line decisions and has been associated with reduced anastomotic leak rates in some series, although results remain heterogeneous overall, supporting the need for standardization of dose, timing, and signal quantification [5].

Beyond the “functional” fluorescence provided by indocyanine green (ICG), molecularly targeted probes couple a specific ligand or antibody (e.g., anti-carcinoembryonic antigen [CEA], anti-epidermal growth factor receptor [EGFR], or anti-hepatocyte growth factor receptor [c-Met]) to a near-infrared (NIR) fluorophore, enabling the selective enhancement of biologically relevant tissues in CRC. Carcinoembryonic antigen (CEA) is a major target; SGM-101 (anti-CEA) has demonstrated clinical feasibility and the potential to alter intraoperative management [6]. In parallel, EGFR and c-Met expression in CRC underpins the development of corresponding targeted probes, while activatable approaches (e.g., cathepsin-based) and immuno-oncologic targeting (e.g., programmed death ligand 1 [PD-L1]) further expand the field of intraoperative molecular imaging. Although the level of clinical maturity varies across these directions, the available evidence supports the translational value of molecular targeting as a complement to ICG in selected scenarios [7].

Several reviews have described individual applications of fluorescence imaging in colorectal surgery, but the field is now moving from proof-of-concept studies toward selective clinical implementation. The added value of the present review is threefold: first, it integrates recent evidence across the four intraoperative decision points at which fluorescence currently has the greatest clinical relevance; second, it distinguishes established ICG-based uses from emerging tumor-targeted and multimodal strategies; and third, it focuses explicitly on the technical and translational factors that determine reproducibility and future adoption, including dose, timing, optics, quantitative interpretation, and AI-assisted analysis. On this basis, we analyze fluorescence-guided surgery in colorectal cancer through four clinical domains: anastomotic perfusion assessment, lymphatic mapping, intraoperative tumor localization, and metastasis detection.

**Figure 1:**
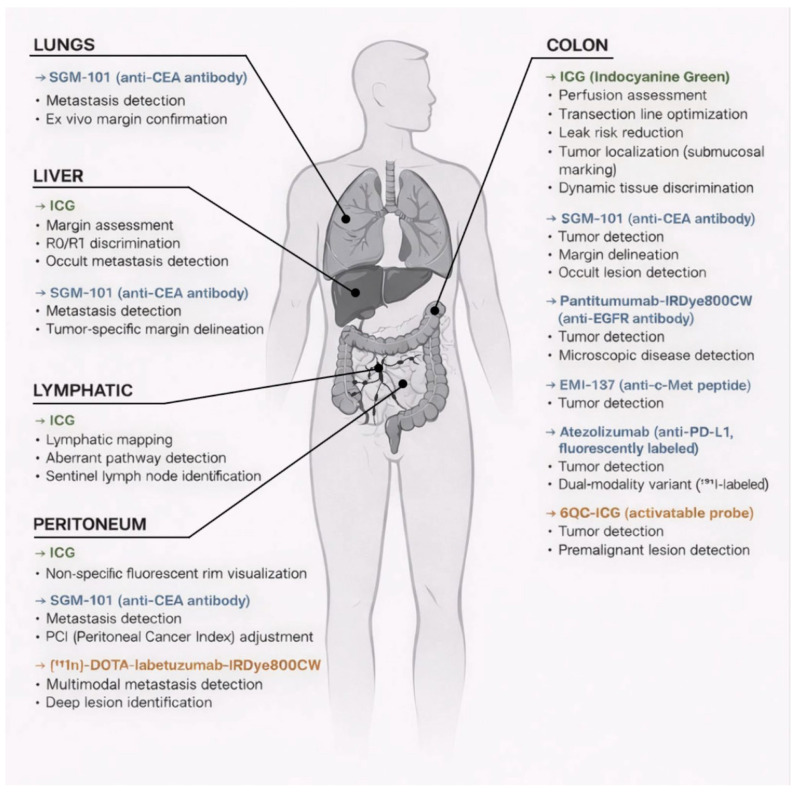
Schematic overview of key near-infrared fluorophores and targeted probes discussed in this review and their primary intraoperative applications in colorectal cancer (CRC), organized by anatomical site. Indocyanine green (ICG) is used mainly for perfusion assessment, lymphatic mapping, and lesion marking, whereas tumor-targeted or activatable probes (e.g., anti-CEA, anti-EGFR, anti-c-Met, anti–PD-L1, and cathepsin-activated agents) aim to improve tumor/margin delineation and metastasis detection; multimodal CEA-targeted tracers enable combined preoperative SPECT/CT and intraoperative fluorescence/gamma-probe guidance. Color coding: green indicates ICG/non-targeted functional fluorescence, blue indicates tumor-targeted fluorescent probes, and orange indicates activatable and/or multimodal probes. Abbreviations: CEA, carcinoembryonic antigen; EGFR, epidermal growth factor receptor; PD-L1, programmed death ligand 1; SPECT/CT, single photon emission computed tomography/computed tomography; PCI, peritoneal cancer index.

An overview of the main fluorophore and targeted-probe classes discussed in this review, along with their principal intraoperative applications across anatomical sites, is provided in Figure 1.

## 2. Materials and Methods

This narrative review was based on a structured search of the PubMed database, employing a predefined combination of free-text terms and Boolean operators. The search strategy included terms related to colorectal cancer (“colorectal cancer”, “colorectal neoplas*”, “colorectal tumo*”, “colonic tumo*”, “rectal tumo*”) combined with keywords referring to fluorescence-guided surgery (“fluorescen* AND surg*”, “image-guided surgery”, “indocyanine green”, “ICG”, “intraoperative fluorescence”, “near-infrared imaging”, “NIRF”). The initial search covered studies published between 2019 and 2025, while earlier articles were considered if they provided landmark evidence or essential background for subsequent developments.

Additional relevant publications were retrieved by cross-referencing the bibliographies of related reviews, in order to capture studies not identified in the primary PubMed search. Selection was not based on a predefined number of articles; instead, inclusion focused on relevance to the major clinical domains of intraoperative fluorescence in colorectal surgery: (i) anastomotic perfusion assessment, (ii) lymphatic mapping, (iii) intraoperative tumor localization, and (iv) detection of metastatic disease.

The review encompassed both preclinical and clinical peer-reviewed publications, including observational studies, randomized and controlled clinical trials, comparative and multicenter studies, and phase I–IV trial protocols.

This narrative approach was selected not only to summarize the results but also to clarify where the evidence is already clinically actionable and where it remains exploratory. Accordingly, throughout this review, we distinguish prospective interventional evidence from observational data and interpret findings in light of implementability, protocol standardization, and the need for objective fluorescence assessment.

## 3. Results and Discussion

*Overview and Organization of the Evidence*

To improve readability, the evidence is organized by clinical question rather than by tracer alone. Within each domain, prospective interventional studies are discussed first, followed by observational series and then a focused synthesis of limitations, sources of heterogeneity, and translational implications. This structure helps distinguish areas supported by higher-level evidence from those that remain primarily hypothesis-generating.

We begin with anastomotic perfusion assessment—the most established use of indocyanine green (ICG) fluorescence angiography—before proceeding to lymphatic mapping, tumor localization, and metastasis detection. For clarity, an overview of the main intraoperative applications, advantages, disadvantages, limitations, and translational perspectives across these domains is provided in Table 1.

## 4. Perfusion Assessment

Indocyanine green fluorescence angiography (ICG-FA) currently represents the most established clinical application of fluorescence in colorectal surgery, being used to evaluate tissue perfusion and reduce the risk of anastomotic leak (AL) by guiding adjustment of the transection line when necessary. For clarity, we first summarize prospective interventional evidence (randomized and non-randomized), then observational/real-world studies, followed by a focused discussion of limitations, sources of heterogeneity, and implementation/future directions.

### 4.1. Prospective Interventional Evidence

Prospective randomized and controlled data suggest that ICG-FA can reduce AL in selected settings, although results are not uniformly consistent across trials and clinical settings. In the EssentiAL randomized clinical trial, Jun Watanabe et al. reported a statistically significant decrease in AL (7.6% in the ICG-FA group vs. 11.8% in the control group, *p* = 0.041) [8]. At the level of aggregated randomized evidence, a recent systematic review and meta-analysis of randomized controlled trials reported a lower overall risk of AL with ICG fluorescence imaging (risk ratio [RR] = 0.54; 95% CI 0.35–0.84), with low heterogeneity (I^2^ = 8.6%). Notably, the pooled benefit was largely driven by lower-grade leaks, whereas higher-grade leaks showed less consistent differences across trials [5].

### 4.2. Observational and Real-World Evidence

In observational and propensity score-matched cohorts, ICG-FA has frequently been associated with a numerical reduction in AL, although statistical significance is variably achieved depending on sample size and protocol [9,10,11,12,13,14,15,16,17,18]. With respect to the modification of the transection line, multiple studies [9,10,11,12,13,14,15,16,19,20,21] reported that, in cases where fluorescence-guided re-resection was undertaken, no subsequent anastomotic leaks occurred, suggesting a potential protective effect of this maneuver. Nevertheless, in the study by Jun Watanabe et al. [8], a single case of AL was reported despite alteration of the operative plan, indicating that the risk may be reduced but not completely eliminated. A single case of leak was also mentioned in the cohort of patients undergoing right hemicolectomy with complete mesocolic excision (CME) in the series by Christoph Marquardt et al. [10]. These divergent results may reflect variability in surgical technique (type of resection, level of the anastomosis) as well as patient-specific factors such as comorbidities.

### 4.3. Discussion: Limitations and Sources of Heterogeneity

Although anastomotic perfusion is an essential factor in the development of leaks, multiple studies have shown that their etiology is complex and multifactorial. Dinallo et al. [19] used SPY-Q software in conjunction with the SPY Elite system for quantitative fluorescence analysis (0–256 units), establishing a threshold for suboptimal perfusion at <40. However, patients who developed leaks exhibited values between 50 and 100, suggesting that apparently adequate perfusion does not entirely exclude the risk of AL. This observation is supported by the multicenter study by Pernille O. Larsen et al. [22], in which patients with AL frequently had associated comorbidities, such as cardiovascular disease, diabetes mellitus, and nodal metastases, confirming the multifactorial nature of the complication. Thus, ICG-FA should be regarded as a complementary intraoperative decision-support tool rather than a definitive or standalone predictor.

Another investigated direction concerned the impact of inferior mesenteric artery (IMA) ligation type on colonic perfusion. In a prospective study, Han et al. [23] showed that although initial parameters suggested an advantage for low tie, the differences disappeared after standardization. Multivariable analysis indicated that low perfusion was associated with an increased risk of AL, whereas the type of ligation did not significantly influence its incidence. These results, however, raise important questions regarding both methodology and clinical interpretation. First, the disappearance of differences after standardization suggests that the apparent benefits of low tie may represent methodological artifacts rather than true clinical advantages, calling into question the validity of certain surgical practices derived from previously published studies. Moreover, the apparent contradiction between the association of low perfusion with increased AL risk and the lack of impact of ligation type suggests that determinants of tissue perfusion are more complex than the simple preservation of collateral circulation through low tie. Intrinsic vascularization of the colonic segment, the patient’s cardiovascular status, and tissue quality may exert a greater impact than the surgical technique per se.

The analyzed studies also present a series of limitations. One of the most important is the subjective nature of perfusion assessment, which in most cases relies on the surgeon’s visual appraisal in the absence of objective criteria or predefined thresholds, resulting in high interobserver variability [22]. Although various research groups have tested quantitative analysis algorithms and even AI systems for interpreting ICG fluorescence [19,22,23,24] most of these approaches remain experimental or have been applied only retrospectively. Therefore, the development and implementation of integrated, real-time decision-support tools are needed to support intraoperative decisions regarding the selection of the transection line and optimization of the anastomosis. Beyond software-based methods, Darwich et al. [25] investigated a different approach, using O2C^®^ spectrophotometry to measure blood flow in colonic segments visually demarcated by ICG fluorescence during laparoscopic low anterior resections (LAR). The authors showed that areas with “optimal” fluorescence consistently exhibited flow values above the threshold of 164 arbitrary units (AU), whereas “sufficient” and “absent” areas had significantly lower values, confirming the method’s reproducibility. However, the leak incidence (5.4%) did not correlate with these values, suggesting that although useful for objectifying perfusion, spectrophotometry requires further validation before it can be considered a reliable clinical predictor.

Another limitation is the small size and design of available studies, most being single-center, retrospective, or based on historical cohorts and small samples, which reduces statistical power and consequently limits generalizability. To overcome these limitations, multicenter, prospective trials with large cohorts are required—similar to that conducted by Jun Watanabe et al. [8], to enable robust validation of the impact of ICG-FA on tissue perfusion and the incidence of anastomotic leaks.

Pernille O. Larsen et al. [22] emphasize an important aspect: perfusion is currently evaluated only on the serosal surface, even though the mucosa is considerably more vulnerable to ischemia. In this context, it would be of interest to investigate the impact of trans-anal ICG-FA, which could provide additional information regarding mucosal perfusion.

Another limitation of the studies is the lack of standardized protocols for quantitative fluorescence assessment. Hong-min Ahn et al. [26] attempted to develop an optimal protocol by analyzing, in in vitro experiments and a prospective clinical cohort, extrinsic factors that may influence measurement results. They recommend maintaining a constant camera-to-tissue distance of 4–5 cm, using fluorescence-dedicated modes (Spectra A for xenon sources and ENV for laser), and switching off ambient lighting for extra-abdominal assessments. This protocol can be applied in clinical practice but requires additional validation to be accepted as a universal standard and to allow the definition of reproducible quantitative thresholds. Regarding ICG dose and timing, the study by Pernille O. Larsen et al. [22] reported that using 7.5 mg compared with 15 mg did not significantly alter the results; however, this finding cannot be considered definitive in the absence of broader methodological and clinical consensus, and further studies dedicated to this aspect are warranted.

Lastly, differences are evident in the literature regarding the timing of ICG perfusion assessment: some studies [8,9,11,12,17,19,21,23] performed a single evaluation before constructing the anastomosis, whereas others [10,13,14,15,16,18,20] evaluated perfusion both pre- and post-anastomosis. Given that stapled suturing can cause mucosal injury, it would be of interest to investigate in the future whether this double fluorescent check is associated with additional benefits in reducing leak risk.

### 4.4. Implementation and Future Directions

Across the available studies assessing perfusion with ICG fluorescence angiography, no intraoperative complications or adverse reactions related to ICG have been reported, further confirming its excellent safety profile. The only absolute contraindication is a known hypersensitivity to indocyanine green or its excipients. From an economic standpoint, the technique appears cost-neutral: in the study by Su et al., hospitalization costs did not differ significantly between the ICG and control groups (9661.9 ± 987.7 USD vs. 9520.0 ± 854.4 USD, *p* = 0.291) [20]. Moreover, a recent cost analysis model by Liu et al. reported an average saving of 192 USD per case with ICG fluorescence angiography. The technique remained cost-effective when the baseline leak rate exceeded approximately 5%, the cost per use was below 600 USD, or the cost of an anastomotic leak surpassed 5600 USD [27].

Despite this favorable safety and economic profile, ICG fluorescence has not yet been universally adopted for colorectal resections. The current barriers are primarily practical rather than biological and align with the limitations outlined above, and include heterogeneity in dosing and timing protocols, lack of universally accepted quantitative thresholds (with most assessments still visual and operator-dependent), variability in NIR imaging systems across centers, and the need for additional operative time and training. Until these methodological aspects are standardized and validated through large multicenter cost-effectiveness trials, routine universal implementation cannot yet be fully justified, despite the evident safety and clinical potential of the technique.

ICG-FA remains a promising intraoperative adjunct for optimizing surgical decision-making and reducing the risk of anastomotic leakage, and the validation of standardized, quantifiable protocols will likely be decisive for its broader universal adoption. We next address lymphatic mapping, where fluorescence is used not to assess perfusion but to visualize lymphatic drainage pathways and nodal basins.

## 5. Lymphatic Mapping

Indocyanine green near-infrared (ICG-NIR) fluorescence enables intraoperative mapping of lymphatic drainage in colorectal cancer, facilitating extended dissection, identification of drainage variants, and exploration of the sentinel lymph node (SLN) concept. As in the previous section, evidence is summarized first for prospective studies and then for observational/real-world series, followed by a focused discussion of technical variability and future directions.

### 5.1. Prospective Interventional Evidence

A consistent finding across clinical studies is the ability of ICG-NIR fluorescence to reveal additional lymph nodes that are not apparent under conventional white-light visualization. In a randomized, prospective study [28], the mean total number of excised lymph nodes (D1–D3 stations) was significantly higher in the ICG group than in the non-ICG group (28 vs. 19, *p* = 0.01), a difference confirmed at each analyzed station. For example, at the D3 station an average of two additional nodes were identified (*p* = 0.03). Moreover, Dario Ribero et al. showed that the use of fluorescence led to extension of the D3 dissection by identifying additional nodes in 6 of 70 patients. In addition, for low rectal cancer the context is particular; in Europe, lateral pelvic lymph nodes (LPLN) are considered aberrant stations outside the limits of total mesorectal excision (TME) and are resected selectively only, whereas in Japan their dissection is part of standard practice [29]. In this setting, study 23 showed that the use of ICG allowed excision of a significantly higher number of LPLN (11.5 ± 5.9 vs. 7.1 ± 4.8; *p* = 0.017). These results support the idea that fluorescence not only facilitates visualization but may effectively increase the yield of nodal dissection; however, it remains to be established to what extent this surplus of identified nodes translates into concrete oncologic benefits.

Although ICG-NIR functions as a marker of lymphatic flow, the technique does not reflect the tumoral status of the node. In the study conducted by Jinliang Wan et al., no statistically significant difference was found between the ICG and non-ICG groups in the proportion of positive nodes among those excised (*p* = 0.386), suggesting that fluorescence does not reliably correlate with the presence of metastases [28]. Similar results were reported in another study [29], in which uptake at the D3 station was not associated with pN status (Fisher’s exact test, *p* = 1.00). In a larger study [30] analyzing 1017 nodes, metastases were twice as frequent in non-fluorescent nodes (OR 2.15; 95% CI 1.08–4.27; *p* = 0.03), with the observation that when >90% of nodal volume was invaded, the fluorescent signal disappeared completely. This biological limitation was also investigated quantitatively by Ying Cao et al. [31] using the FLI-10B camera. The mean fluorescence intensity was similar between benign and metastatic nodes, without clinically relevant differences. Receiver operating characteristic (ROC) analysis showed that the signal-to-background ratio (SBR) provided acceptable sensitivity (80%) but low specificity (51.4%), with a positive predictive value of only 19%. Thus, overall, these findings suggest that ICG fluorescence does not provide reliable discrimination between benign and metastatic nodes. From a translational perspective, overcoming this limitation would require the development and clinical validation of targeted molecular tracers (such as anti-CEA or anti-EGFR) to transform lymphatic mapping from a purely anatomic marker into a tool with true oncologic value.

### 5.2. Observational and Real-World Evidence

Another contribution of fluorescence is the ability to highlight unusual drainage pathways and nodes located outside the conventional dissection field. In the GREENLIGHT study [29], fluorescent-positive extra-basin nodes (median: two per patient) were identified in 29 of 70 patients and excised using a “berry-picking” technique; none were metastatic, and staging was therefore not altered. In total, of the 102 aberrant nodes excised, most were para-aortic (39; 38.2%), predominantly on the right side. Similar results were reported by Caprioli et al., in which extension of dissection was required in 19 of 32 cases due to fluorescent-positive nodes, while histopathologic analysis of the 50 additional nodes identified no metastases. The described locations included the origin of the middle colic artery, the left colic artery, the level of the superior mesenteric artery, the root of the inferior mesenteric artery (IMA), para-aortic sites, and the mesenteric veins [32]. Other studies [28,33,34] likewise reported identification of extra-basin nodes, although with less consistent results.

Two important aspects regarding extra-basin nodes merit emphasis. The first concerns the patients’ initial staging: in a study by Michela Caprioli et al. [32], most cases were T3–T4 tumors, and in GREENLIGHT [29] more than 85% were at least T2. This is relevant given that isolated metastases in aberrant nodes are rare (≈1%) and occur almost exclusively in T3–T4 cancers, as demonstrated by Saha et al. [35]. The second aspect concerns the therapeutic importance of these nodes. Although in the studies discussed here no aberrant node proved metastatic, the study by Jaram Lee et al. reported para-aortic lymph node (PALN) metastases in 7.2% of patients, more than a quarter of which were discovered only intraoperatively [36]. Moreover, when an R0 resection was achieved, 5-year survival was comparable to that of patients without PALN metastases (90.0% vs. 82.2%; *p* = 0.896). Overall, these data indicate that fluorescence can map unusual lymphatic pathways, yet their oncologic value remains uncertain; nonetheless, from this perspective, even if current results support a predominantly anatomic role, in certain selected contexts, dissection of aberrant nodes may acquire clinical relevance.

Although the available literature is limited, experience with ICG-NIR for SLN identification in colorectal cancer has been explored in several clinical series, with promising yet heterogeneous results. Unlike classical technologies (blue dye, radioisotopes), the application of ICG in the colorectal SLN technique remains insufficiently investigated. In the study by Daan J. Sikkenk et al. [37], the SLN detection rate was 100% (mean 2.3 SLN per patient; diameters 1–13 mm). In total, metastases were identified in 2 of 10 patients, located in the SLN itself, with no false-negative results; the negative predictive value was therefore 1.0. In contrast, in another series [38], the identification rate was 56.5% (13/23), which the authors attributed to the learning curve and inclusion of cases performed via open surgery. Although in 7/13 cases the correlation between fluorescent drainage and metastases was adequate, there were three false-positive SLN, limiting the accuracy of the method. These preliminary results suggest a real potential for ICG in the SLN technique; however, current evidence is insufficient to support clinical implementation. Larger, prospective multicenter studies are needed to validate ICG in colorectal SLN mapping and to establish a standardized protocol.

### 5.3. Discussion: Limitations and Sources of Heterogeneity

For correct interpretation of the data, it is essential to discuss the main limitations of the analyzed studies, as these influence both the robustness of the conclusions and the clinical applicability of the technique. A major but potentially correctable limitation is the lack of a standardized protocol for ICG injection, which generates considerable variability across studies and complicates cross-study comparisons. Protocols differ in several respects. First, the concentration used: although 25 mg/10 mL is frequently employed [28,30,32,38], variations exist such as 0.5 mg/mL [29], 0.1 mg/mL [39], 25 mg/5 mL [37], or 25 mg/30 mL [31]. Second, the timing of injection can be preoperative or intraoperative. Most studies opted for preoperative administration [28,29,31,32,39], with more robust data in the study by Dario Ribero et al., in which no statistically significant difference was observed between injection at 24 h and at 72 h preoperatively (*p* = 1.00), with comparable visualization rates at the D3 stations (91.8% vs. 95.3%) [29]. This observation is supported by Caprioli [32], who noted that only administration more than 7 days beforehand leads to fading of fluorescence and reduced accuracy of the technique. Other studies explored intraoperative injection—laparoscopic [32,37], after laparotomy [40] or transanal [39]. Third, the mode of injection varied and included colonoscopic [28,29,30,32,37], direct laparoscopic [32], transanal [39], or even intravenous [31]. Regarding the number and volume of peritumoral injections, the usual approach was injection at 2–4 points, with volumes varying among studies (e.g., 2–3 mL per point [28], 1.5 mL in 25, 1 mL [37]). The injection site was also variable: submucosal for colonoscopic administration and subserosal for laparoscopic administration; nevertheless, in the study by Caprioli [32], no significant differences were observed in the number of fluorescent-positive nodes between the two approaches (*p* = 0.5). Another debated aspect was the need for submucosal elevation before injection. In some studies [28,30,32] it was used to prevent ICG extravasation, whereas in others [29] it was avoided to prevent dilution of the substance. Overall, these discrepancies illustrate how many technical factors can influence results. Thus, it is absolutely necessary to develop a standardized, rigorous, and universally adopted protocol to allow comparability between studies and to provide a solid foundation for clinical validation of the technique.

The limited design of available studies represents a major restriction on data interpretation. Most are single-center, prospective, and largely observational, with relatively small samples and consequently limited statistical power. Only a single study had a randomized controlled design, underscoring the absence of high-level evidence. Clinical validation of fluorescence would require multicenter, randomized studies with large cohorts and well-defined inclusion criteria, enabling comparative evaluation of oncologic outcomes and long-term safety.

Another major limitation of the literature is the lack of standardized follow-up, which is essential for evaluating the true oncologic impact of the technique. In the study by Si-Cheng Zhou et al. [39], although there were no residuals or recurrences in the ICG group and two cases of early recurrence (at 10 days and 3 months) in the control group, monitoring was not uniform. In another study [32], patients were followed for three years without nodal recurrences, but such data are rare. Integration of systematic, long-term follow-up remains indispensable for clinical validation of fluorescence in oncologic lymphadenectomy.

A further limitation is the lack of an objective evaluation of fluorescence. In all studies, assessment was subjective and performed visually by the surgeon, with the exception of the study by Ying Cao et al. [31], who used a dedicated quantification system (FLI-10B). Although this approach enabled standardized measurement of signal intensity, the method remains isolated and, in the absence of integrated tools applicable in real time, interpretation continues to depend predominantly on operator experience.

### 5.4. Implementation and Future Directions

Clinical evidence to date indicates that the use of ICG-NIR fluorescence for lymphatic mapping in colorectal cancer is consistently safe and well tolerated, with no intraoperative complications or adverse reactions attributable to the dye. The only absolute contraindication remains a known hypersensitivity to indocyanine green or iodine, while additional conditions such as pregnancy, severe hepatic or renal impairment, or thyroid hyperfunction are regarded merely as precautionary. From an economic perspective, no dedicated cost analyses have been published, yet the technique is uniformly described as low-cost and readily implementable across different surgical settings.

Despite this favorable safety profile, the current barriers to widespread adoption are methodological rather than biological, reflecting the same limitations identified across studies: absence of standardized protocols, reliance on subjective visual interpretation, small and heterogeneous cohorts, and lack of long-term oncologic validation. Until these aspects are addressed through large, quantitative multicenter trials, ICG-guided lymphatic mapping should be considered primarily an anatomic and navigational adjunct, whose oncologic value remains to be conclusively demonstrated. We next focus on tumor localization, including timing-dependent ICG behavior and emerging targeted probes designed to increase oncologic specificity.

## 6. Tumor Localization

Intraoperative fluorescence, including emerging applications based on molecular agents, has established itself as a central strategy for tumor localization and margin assessment, with current directions moving beyond the demonstrative stage and focusing on method refinement and leveraging the signal in standardizable clinical contexts. In this section, we first summarize ICG-based clinical evidence (including timing-dependent behavior and comparison with contrast-free optical adjuncts), then review targeted/activatable probes, followed by a discussion of limitations and translational directions.

### 6.1. ICG-Based Clinical Evidence

In particular, for indocyanine green (ICG), attention has shifted to temporal analysis of the signal, optimization of administration strategies, and comparison with other optical techniques.

A key aspect investigated recently is the distinct geotemporal behavior of ICG fluorescence in malignant tissues compared with benign or normal tissues during intraoperative assessment. In a prospective study published in 2021 [40], in vivo dynamic analysis showed that malignant colorectal tumors reached higher peak intensities (202 ± 35.7 g.u.) than benign lesions (165.8 ± 11.7 g.u., *p* = 0.02) and normal tissue (169.6 ± 12.8 g.u., *p* = 0.04). They were characterized by a slower rise gradient (*p* = 0.039), a longer time to peak (*p* = 0.03), and delayed clearance, with persistence of fluorescence beyond 100 s after the maximum (*p* = 0.03). In contrast, benign lesions exhibited a more rapid post-peak decrease in intensity (*p* = 0.046). Conversely, static ex vivo measurements performed a few hours after ICG administration (“spot FI”) failed to discriminate between cancer and normal tissue (*p* > 0.05), with signal overlap limiting the diagnostic value of this approach. Subsequently, in 2024, the geotemporal nature of ICG fluorescence was also investigated by Hardy et al. [41], who analyzed the signal at early (<15 min after intravenous administration) and late (>2 h) intervals. In the early phase, macroscopic assessment demonstrated that malignant colorectal tumors displayed significantly more intense fluorescence than benign lesions (121.53 ± 11.25 vs. 102.55 ± 4.91 g.u., *p* < 0.001), with heterogeneous signal distribution (pixel-intensity SD: 18.87 ± 6.91 vs. 7.85 ± 3.44 g.u., *p* < 0.001), reflecting stromal and vascular features of the tumor microenvironment. This “dynamic early phase NIR imaging” thus proved most useful for malignancy–benign differentiation. In contrast, at >2 h after injection, macroscopic differences between cancer and healthy tissue were no longer significant (40.6 ± 22.2 vs. 29.7 ± 20.0 g.u., *p* = 0.153), limiting the diagnostic value of a single static evaluation. Microscopic analysis showed that, early on, fluorescence accumulates predominantly in the stroma and vascular structures, with a significant stroma–gland difference (*p* < 0.001), whereas later it penetrates intracellularly into malignant glands, differentiating them from benign glands (*p* < 0.001), although peritumoral inflammation sometimes produced more intense uptake than the tumor, with potential false-positives. Overall, these results indicate that the clinical utility of ICG fluorescence is closely dependent on the temporal window of assessment, with early dynamic analysis demonstrating superior value compared with static assessment. This finding underscores both the current limitations of the method and the need for its standardization and validation to enable reproducible clinical implementation.

Beyond perfusion assessment, dynamic ICG fluorescence analysis has also been explored for intraoperative tissue characterization. In 2021, Cahill et al. demonstrated that dynamic fluorescence analysis integrated into an artificial intelligence (AI) system can distinguish between benign lesions, malignant lesions, and normal mucosa, with a mean accuracy of 86% and a sensitivity of 100% at the level of the entire lesion [24]. In 2022, Jonathan P. Epperlein et al. showed that machine learning algorithms based on simple parameters extracted from time–fluorescence curves (particularly wash-out slopes) can robustly differentiate benign from malignant lesions, achieving an accuracy of approximately 95% [42]. In 2023, Hardy et al. further advanced this direction by applying machine learning algorithms to intraoperative ICG fluorescence angiography recordings; malignant, benign, and healthy tissues could be differentiated with excellent accuracy (area under the receiver operating characteristic curve [AUC-ROC] > 0.9) [43]. Collectively, these results support the concept of a “digital biopsy,” although routine clinical implementation requires validation in large, multicenter studies.

Building on these observations regarding fluorescence dynamics, another essential aspect investigated recently is the optimal timing of preoperative ICG administration. Tetsuta Satoyoshi et al. [44] showed that submucosal injection of ICG (0.5 mg at two points) constitutes a safe and reliable method for marking colorectal tumors, being intraoperatively detectable in 100% of cases when performed ≤ 6 days before surgery. Visibility falls significantly to 60% if marking is performed at 7–9 days and disappears completely after ≥10 days (*p* < 0.0001). This strict time dependence highlights a major practical limitation and underscores the need for standardized protocols to ensure reproducible clinical performance.

### 6.2. Targeted and Activatable Probes

The expanding use of ICG has prompted comparison with advanced optical technologies, such as hyperspectral imaging (HSI), to evaluate relative advantages and complementarity. Boris Jansen-Winkeln et al. [45] showed that both techniques localize the perfusion frontier almost identically (R = 0.991; *p* = 0.704), with concrete intraoperative impact: in 33.3% of patients, the transection line was modified. ICG fluorescence angiography (ICG-FA) provides a clearer and narrower perfusion frontier (0.31 cm vs. 0.66 cm for HSI, *p* < 0.001) but requires evaluation at the optimal time, around 30 s after injection, to avoid overestimation of perfusion. HSI, although producing a more diffuse frontier, does not require contrast and provides multiple oxygenation and perfusion parameters. From a translational perspective, integrating the two technologies—ICG-FA for rapid decision-making and HSI for confirmation and additional detail—may represent a potentially superior strategy for intraoperative tumor localization and margin definition, although further clinical validation is required.

Beyond ICG, emerging directions in tumor localization involve probes with higher molecular specificity that selectively highlight epithelial components (CEA, EGFR), oncogenic surface receptors (c-Met), functional features of the microenvironment (activatable cathepsin probes), or immunologic determinants (PD-L1). The level of maturity ranges from clinical data (SGM-101) to preclinical/early phase studies (panitumumab-IRDye800CW, EMI-137, 6QC-ICG, atezolizumab), collectively delineating the translational pathway toward personalized intraoperative guidance anchored in CRC tumor biology.

SGM-101, a fluorescently labeled anti-CEA antibody, targets carcinoembryonic antigen (CEA), frequently overexpressed in colorectal cancer and correlated with an unfavorable prognosis [46], and is among the first molecular probes evaluated in intraoperative clinical studies. In a multicenter phase I/II study of 37 patients [47], intravenous administration of a 10 mg dose four days preoperatively provided the best tumor contrast (tumor-to-background ratio [TBR] 1.9; *p* = 0.019) and solid diagnostic performance: sensitivity 96%, negative predictive value 94%, and overall accuracy 78%. Notably, fluorescence was absent in patients with a pathologic complete response after neoadjuvant therapy, suggesting a direct correlation with tumor viability. The major limitation was limited signal penetration (≈1 cm), rendering some lesions detectable only ex vivo on the resected specimens. Ultimately, the clinical impact was relevant. In 24% of patients, fluorescence led to justified changes in the surgical plan by identifying additional lesions or avoiding extensive resections. From a translational standpoint, SGM-101 is shaping up as the first standardized molecular agent with potential for clinical implementation in fluorescence-guided CRC surgery.

Panitumumab-IRDye800CW leverages elevated EGFR expression, present in approximately 60–80% of colorectal cancers [48], and has been evaluated preclinically as a targeted imaging agent. In a prospective study in nude mice xenografted with CRC lines of differing genetic status (LS174T, KRAS-mutant; Colo205 and SW948, KRAS wild-type) [49], intravenous administration of 200 μg panitumumab-IRDye800CW generated significantly higher tumor-specific contrast than control IgG (*p* < 0.05). Over time, TBR remained consistently superior in all models (e.g., Colo205: 5.78 vs. 2.52; *p* < 0.05), peaking at day 10 (LS174T: 10.8; Colo205: 12.5). Interestingly, the SW948 line exhibited the highest EGFR expression by IHC and Western blot yet a more modest TBR (5.7), underscoring the influence of tumor architecture and vascularization on uptake. In addition, fluorescence enabled detection of submillimetric tumor fragments (~1 mg, *p* < 0.05), confirming sensitivity for microscopic disease. These results indicate that panitumumab-IRDye800CW may be used not only as an anti-EGFR therapy but also as a high-accuracy imaging vehicle in CRC. From a translational perspective, its provision of specific contrast even in the KRAS-mutant model suggests that imaging applicability is not conditioned by RAS status, unlike anti-EGFR therapy. In parallel, the recent FDA approval of panitumumab plus sotorasib for mCRC with KRAS G12C mutation confirms the continued clinical relevance of the antibody in a broader patient spectrum, reinforcing the rationale for its exploratory use as an imaging vehicle.

EMI-137 was designed to target the c-Met receptor, implicated in colorectal cancer progression and metastasis, and associated with an unfavorable prognosis [50], and has been investigated both preclinically and in a feasibility clinical study. Gemma R. Armstrong et al. [51] demonstrated in vitro specificity, where c-Met knockdown by siRNA in HT-29 cells completely abolished the fluorescent signal. In in vivo models, in HT-29 xenografts in BALB/c nude mice, administration generated a clear tumor signal as early as one hour post-injection, persisting up to 6 h, with predominantly renal biodistribution and rapid clearance. In the phase IIa clinical stage, in nine patients undergoing laparoscopic resection, a single 0.13 mg/kg dose was well tolerated with no agent-related adverse events. However, clinical performance was modest: only 44% of patients showed visible intraoperative tumor contrast, and none of the histologically positive nodes (15/15) were fluorescently detected. Immunohistochemical analysis confirmed increased c-Met expression in tumors and positive nodes, but also in normal colonic mucosa, generating background fluorescence and reducing the signal-to-noise ratio. Thus, although EMI-137 validates anti-c-Met imaging specificity, intraoperative laparoscopic application is currently limited by this physiological intestinal c-Met expression and the resulting background signal. From a translational perspective, its utility may be greater in colonoscopy—where mucosal visualization is direct—or after optimizing administration timing and optical systems to increase contrast and enable nodal detection.

6QC-ICG is an activatable probe that becomes fluorescent upon peptide-linker cleavage by cathepsins, proteolytic enzymes hyperactive in the colorectal tumor microenvironment [52], offering a functional approach for intraoperative detection of malignant tissue. Joshua J. Yim et al. [53] demonstrated the feasibility of this strategy in preclinical CRC models. In Apc^Min/+ mice (spontaneous intestinal carcinogenesis), administration of 2.3 mg/kg produced high contrast (TBR 3.9; *p* < 0.0001), with signal visible also in lymphoid structures rich in macrophages, confirming microenvironment targeting. In Apc^Pirc/+ rats (intestinal polyposis), NIRF endoscopy highlighted polyps as small as 2 mm, with TBR 2.1 and CD68+ histologic correlation, suggesting utility for subtle lesion detection. In the porcine APC^1311/+ model (anatomy comparable to humans), at 1.0 mg/kg, NIRF-guided biopsies achieved 100% Se/Sp, with histologically validated TBR thresholds for differentiation (≥2.1 for dysplasia; <1.2 for normal tissue). Lesions as small as 400 μm were detected, and even against a severe inflammatory background (AOM-DSS), the tumor contrast remained significant (TBR 1.81; *p* = 0.0011). Overall, 6QC-ICG highlights the advantages of activatable probes, which fluoresce only in proteolytic environments, providing practical contrast over short intervals (12–24 h). From a translational perspective, this functional approach overcomes limitations of single-receptor targeted agents, outlining a promising candidate for clinical trials of fluorescence-guided colonoscopy with direct impact on miss-rate reduction and optimization of targeted biopsies in high-risk patients.

Atezolizumab, fluorescently or radioactively conjugated, is under investigation as a targeted imaging agent for PD-L1, whose expression is present in ~40% of colorectal cancers at the level of tumor cells and up to 95% when immune cells are included [54], with higher prevalence in MSI-H/dMMR subgroups. Linhan Zhang et al. tested this approach in a preclinical study in Balb/c nude mice xenografted with CRC lines exhibiting different PD-L1 expression: RKO (high) and HCT8 (low) [55]. In vitro, uptake of ^131I-atezolizumab was 62.1% in RKO cells versus 2.35% in HCT8, confirming specificity. In vivo, fluorescent labeling produced a visible tumor signal at 24–72 h, with attenuation at 168 h, whereas nonspecific IgG persisted longer through the enhanced permeability and retention (EPR) effect. Ex vivo analysis showed higher signal-to-background ratios for RKO compared with HCT8 (tumor-to-skeletal muscle ratio: 3.24× vs. 2.07×). Complementarily, the radioactive variant (^131I-atezolizumab) significantly reduced tumor volume compared with control (259.6 vs. 510.8 mm^3^; *p* = 0.007). However, it did not outperform nonspecific IgG (*p* = 0.137), indicating that the observed signal is substantially influenced by passive accumulation via the EPR effect. In addition, the IgG group exhibited major toxicity (>20% weight loss), underscoring the relevance of specific targeting for an acceptable safety profile. These results demonstrate the feasibility of using fluorescently or radioactively labeled atezolizumab for targeted CRC detection, with clear differentiation between PD-L1-high and PD-L1-low lines. From a translational perspective, anti-PD-L1-based dual-modality approaches can contribute to characterizing tumor heterogeneity and identifying margins in CRC; however, robust clinical validation and standardization of the optimal evaluation timepoint are required to translate this strategy into a reproducible clinical tool.

### 6.3. Discussion: Limitations and Sources of Heterogeneity

Overall, these data confirm that emerging markers constitute a research field with substantial potential, offering prospects for individualizing fluorescence-guided surgery in CRC; at the same time, they warrant continued rigorous, standardized clinical studies to validate their utility and reproducibility in current practice.

At a general level, these studies share common limitations (small sample sizes, heterogeneity of experimental models, and lack of standardization of timing and optical platforms) that restrict immediate clinical applicability. Addressing these barriers through multicenter trials and the development of validated devices will be essential for integrating these agents into routine practice.

### 6.4. Translational Perspectives and Future Directions

Across tumor-localization applications, future progress will depend on harmonizing timing and dosing protocols for ICG-based workflows, establishing reproducible quantitative metrics (e.g., tumor-to-background ratios and time-intensity parameters) across imaging platforms, and validating targeted probes in multicenter studies with clinically meaningful endpoints (margin status, occult lesion detection, and management change) to support broader clinical implementation. Finally, we address fluorescence-guided detection of metastatic disease, where sensitivity/specificity trade-offs and multimodal targeting strategies are particularly relevant.

## 7. Detection of Metastases

Following the established applications in assessing anastomotic perfusion, lymphatic mapping, and intraoperative localization of the primary tumor, near-infrared fluorescence (NIRF) imaging has expanded into a critical domain of oncologic surgery: metastasis detection. In the following subsections, we first summarize observational/real-world evidence for ICG-based NIRF imaging in metastatic CRC, then discuss prospective interventional studies using targeted and multimodal approaches, before outlining key limitations and translational directions across organ sites.

### 7.1. Observational and Real-World Evidence: ICG-Based NIRF Imaging

With regard to hepatic metastases, the essential role of ICG-based NIRF in intraoperative margin characterization should be emphasized, given its direct impact on the likelihood of achieving a complete resection. In the study by Achterberg et al. [56], all lesions that exhibited fluorescence at the protruding rim (7/7) were confirmed as R1 resections on histopathologic assessment. Moreover, three of these lesions also displayed fluorescence in the wound bed; however, the authors considered this sign to be less specific. Conversely, absence of fluorescence at the protruding rim was associated with an R0 resection in eight of nine cases, yielding an overall accuracy of 94% for margin-status prediction. In a larger cohort [57], the use of ICG-based NIRF to guide transection and immediate re-resections significantly increased the R0 rate from 87.4% to 92.4% (*p* < 0.001). A specificity of 90% and a negative predictive value of 92% suggest that the absence of the fluorescent signal correlates strongly with an R0 status, favoring consideration of ICG-based NIRF as a rule-out tool rather than a confirmatory one in this context. From this perspective, the technology provides real support to the surgeon during intraoperative decision-making, offering an objective criterion for extending or maintaining the resection.

Another point of interest is the ability of ICG-based NIRF to reveal additional occult lesions. In the extended series published by Achterberg et al. [57], fluorescence led to the identification of additional lesions in 9.5% of patients, 68.5% of which were subsequently confirmed as metastatic. The sensitivity of this detection was 90%, but the specificity was low (27%), raising the concern of unnecessary resection of benign lesions. The clinical relevance of this observation is reinforced by the report by Achterberg et al. [56], of an occult hepatic metastasis undetected by white light or intraoperative ultrasound but revealed by fluorescence. These results suggest that ICG-based NIRF can increase the sensitivity of hepatic metastasis detection; however, its diagnostic accuracy requires continuous correlation with other intraoperative modalities to avoid excessive therapeutic interventions.

The subjectivity of fluorescent signal interpretation, as described by Hardy et al. [58], also warrants emphasis. To overcome this limitation, the authors explored the integration of artificial intelligence and computer vision for automated fluorescence analysis. Within minutes of ICG administration, their algorithm correctly classified 97.2% of malignant regions of interest (ROIs) and 100% of benign ROIs, while generating two-dimensional maps that facilitated the identification of lesions difficult to visualize in white-light mode. This approach has the potential to standardize fluorescent signal analysis and reduce operator-dependent variability, paving the way for a more objective and reproducible use of the technique.

### 7.2. Prospective Interventional Evidence: Targeted and Multimodal Approaches

Nevertheless, ICG fluorescence does not enable direct visualization of tumor cells, typically generating a peritumoral fluorescent rim rather than a tumor-specific signal. In this context, a major translational direction involves the development of targeted fluorophores capable of binding specifically to tumor antigens such as carcinoembryonic antigen (CEA), which is overexpressed in 98.8% of colorectal cancers [59]. A notable example is SGM-101, a chimeric anti-CEA antibody conjugated to an NIR fluorophore (emission at 705 nm). In the study by Hardy et al. [58], SGM-101 identified 19 fluorescent lesions, 17 of which were subsequently confirmed as malignant, with an overall accuracy of 89%. Immunohistochemistry showed that intense fluorescence correlated with high CEA expression (tumor intensity score [TIS] = 12), whereas borderline signals (TBR ≈ 1.3–1.5) were observed both in malignancies with low CEA expression and in CEA-negative benign lesions, mandating cautious interpretation and potentially stricter positivity thresholds.

Building on these observations, an important translational concept has been proposed: the simultaneous use of two independent spectral channels with complementary functions. The 700 nm channel, corresponding to SGM-101, provides a true tumor map by directly highlighting CEA-positive cells; in parallel, the 800 nm channel, associated with ICG, provides a functional map by delineating the peritumoral rim and areas of altered perfusion or abnormal drainage. In this way, targeted fluorescence confers tumor specificity, while ICG adds functional sensitivity and contributes to margin delineation and detection of occult lesions. Integration of the two approaches could potentially achieve an optimal balance between sensitivity and specificity, with the potential to transform intraoperative detection of hepatic metastases into a standardized, high-accuracy process.

Regarding NIRF detection of colorectal peritoneal metastases, recent research has focused on antibody-based CEA-targeted tracers. In 2020, Schaap et al. [6] investigated whether SGM-101-mediated tumor-specific fluorescence could modify the clinical peritoneal cancer index (PCI) and, implicitly, influence completeness of cytoreduction. Fluorescence altered the PCI in half of the patients (7/14), four of whom were histologically confirmed as having additional metastases. In two patients, the fluorescent signal was false-positive, whereas in one patient fluorescence corrected a clinical suspicion of malignancy, lowering the PCI. On a per-lesion basis (*n* = 103), the technique showed a sensitivity of 98.5% and an NPV of 95.8%, underscoring its ability to exclude metastases. Specificity was more modest (62.2%), indicating a risk of unnecessary resections in benign lesions. Overall, the study showed that SGM-101 can increase detection accuracy and influence surgical decision-making, albeit with a clear limitation due to the risk of false positives, which requires complementary evaluation.

Subsequently, Jean Marie de Gooyer et al. [60] advanced this direction by developing a CEA-targeted dual-label complex, [111In]In-DOTA-labetuzumab-IRDye800CW, combining preoperative SPECT/CT with intraoperative NIR-fluorescence and gamma-probe guidance in a multimodal detection strategy. SPECT/CT detected metastases in 8/10 patients at 10–50 mg doses, especially peritoneal deposits > 10 mm, but underestimated disease extent (PCI 3 ± 3 vs. 10 ± 6 surgically, *p* < 0.001), confirming that its main value lies in patient selection and avoidance of unnecessary laparotomies rather than accurate intraoperative disease quantification. Intraoperatively, NIR achieved a sensitivity of 95% at 10 mg, with a false-positive rate of 16%, increasing to 22% at 50 mg. This limitation justifies the integration of the gamma probe, which is indispensable for detecting deep or non-fluorescent lesions such as retroperitoneal lymphadenopathy or visceral invasion. The multimodal strategy led to changes in the operative plan in approximately 30% of cases, highlighting the practical impact of this approach and highlighting the need for an integrated algorithm that leverages the complementarity of NIR and gamma probing.

In 2025, Aya Darai et al. [61] conducted a confirmatory phase II study to validate prior observations and test the robustness of CEA-targeted multimodality in a new patient cohort. SPECT/CT continued to underestimate PCI (3 ± 2 vs. 14 ± 7; *p* = 0.032), reinforcing its role in staging and patient selection rather than precise assessment of peritoneal extent. Intraoperatively, NIR fluorescence detected 92% of malignant lesions (34/37), with a reduced false-positive rate of 8%, and the gamma probe confirmed complementary value with a radiotracer TBR of 4.4 and the ability to filter nonspecific signals and highlight deep foci inaccessible to fluorescence. This combination enabled the identification of five additional lesions and changes in management in three cases, confirming the practical utility and reproducibility of the strategy. Beyond validation, the study emphasizes the importance of standardizing a sequential multimodal algorithm that includes preoperative SPECT/CT, visual PCI assessment, NIR-based adjustment, targeted resection, and gamma-probe completion for optimal oncologic detection.

Taken together, the analyzed studies demonstrate clear progression from the feasibility of anti-CEA fluorescence to validation of a standardizable multimodal strategy with real potential to transform the surgical management of peritoneal carcinomatosis.

Lastly, concerning pulmonary metastases, the study by R.P.J. Meijer et al. [62] evaluated the feasibility of fluorescence detection by preoperative tracer administration and analysis of the signal both intraoperatively and ex vivo. In a cohort of 13 patients (18 pulmonary metastases), in vivo fluorescence was identified in only 31% of lesions, with a clear correlation to depth from the pleura. No metastasis located > 14 mm from the pleural surface was fluorescent, whereas nearly half of the subpleural lesions were detected. Ex vivo analysis increased the detection rate (47% on resected specimens and 94% on sections), confirming the high specificity of CEA targeting but also the significant limitations of in vivo optical penetration. Clinically, the technique did not reveal previously unknown metastases, but it provided additional information on resection margins (one case with further resection; all specimens R0) and enabled benign-versus-malignant differentiation at the nodal level (three malignant nodes true-positive, two benign nodes true-negative). These results suggest that although the tracer demonstrates robust targeting, intraoperative integration for pulmonary metastases remains limited by technical factors (depth, camera sensitivity), necessitating the development of higher-sensitivity optical systems and careful case selection (e.g., subpleural lesions).

### 7.3. Discussion: Limitations and Sources of Heterogeneity

Most studies were pilot, single-center, with small cohorts and without control arms or randomization. Even the larger multicenter investigations [57,61] remained single-arm, limiting direct comparison with established imaging methods. These methodological features lower the level of evidence and highlight the need for validation in randomized studies and standardized comparative designs with large cohorts and uniform inclusion criteria.

### 7.4. Translational Perspectives and Future Directions

An additional obstacle is the lack of harmonization of experimental protocols. For ICG, variable schemes have been employed, ranging from intraoperative bolus administration (0.05 mg/kg) for perfusion analysis to fixed 10 mg injections 24 h preoperatively for margin assessment [56,57,58]. For SGM-101, the dose has ranged from 5 to 15 mg, with administration intervals of 2 to 6 days preoperatively [6,63]. Moreover, interpretation of the fluorescent signal has not been standardized, spanning subjective visual evaluation and automated AI-based analysis algorithms [58]. This methodological heterogeneity hampers comparability and consistent reproduction of results across centers, underscoring the need to develop a rigorous, uniform protocol.

## 8. Conclusions

Fluorescence-guided surgery in colorectal cancer has progressed from a primarily technical innovation to a selective intraoperative decision-support tool. At present, the most mature application is ICG-based perfusion assessment, whereas lymphatic mapping, tumor localization, and metastasis detection remain more dependent on clinical context, protocol design, and imaging technology. The main contribution of this review is to place these applications on the same clinical-translational continuum: established ICG workflows at one end, and targeted probes, multimodal imaging, quantitative analytics, and AI-assisted interpretation at the other. Viewed in this way, the central challenge is no longer whether fluorescence can generate a useful signal, but how that signal should be acquired, quantified, and integrated into surgical decision-making in a reproducible manner. Broader adoption will therefore require standardized workflows, cross-platform validation, and multicenter studies that use clinically meaningful endpoints.

## Figures and Tables

**Table 1:** Key findings regarding the use of fluorescence-guided surgery in colorectal cancer, including study-design considerations (prospective vs. observational evidence).

**PERFUSION ASSESSMENT**				
**Intraoperative Aim**	**Advantages**	**Disadvantages**	**Limitations**	**Translational Perspectives**
– Assess anastomotic perfusion to optimize transection and reduce anastomotic leak	• Lower anastomotic leak reported in multiple series; supports decision-making • Enables immediate re-resection when fluorescence is poor • Excellent safety; only absolute contraindication is prior ICG/iodine hypersensitivity • Cost-neutral or potentially cost-effective in models • Usable in open, laparoscopic, and robotic workflows; complementary to hyperspectral imaging	• Inconsistent effect across studies, with some neutral trials • Adequate fluorescence does not guarantee zero leaks (multifactorial etiology) • Adds operative time and requires training • Possible over-reliance on subjective visual appraisal	• Prospective evidence: mixed effects across randomized/non-randomized trials; benefit appears protocol-dependent • Observational evidence: numerical AL reductions and transection-line changes are frequently reported, but confounding is possible • Heterogeneous dose and timing across studies • No universally accepted quantitative thresholds; predominantly visual, operator dependent • Platform variability (optics, gain, distance, filters) • Many single-center, small cohorts; typically serosal-side view only • Inconsistent pre- versus post-anastomosis checks; limited linkage to long-term outcomes	• Standardized protocols, cross-platform calibration, and reporting checklists • Real-time quantification of time-intensity curves and AI-assisted interpretation • Multicenter randomized trials powered for leak rates and economics • Hybrid fluorescence angiography plus hyperspectral imaging workflows
LYMPHATIC MAPPING				
**Intraoperative aim**	**Advantages**	**Disadvantages**	**Limitations**	**Translational perspectives**
– Map lymphatic drainage paths and stations; selective sentinel node exploration	• Increases lymph node yield and reveals aberrant drainage that can change the dissection extent • Safe and low barrier to adoption • May improve staging accuracy by reducing missed basins	• With ICG alone, cannot distinguish benign from metastatic nodes • Heterogeneous sentinel node performance across series • Risk of non-oncologic “berry-picking” if the SLN concept is misapplied	• Prospective evidence: improved visualization/nodal yield and mapping of aberrant drainage, but no reliable discrimination of metastatic nodes with ICG alone • Observational evidence: single-center series with heterogeneous protocols and limited long-term oncologic follow-up • No standardized injection protocol for dose, sites, and timing • Predominantly subjective visual assessments • Small, single-center cohorts with heterogeneous case mix • Limited oncologic follow-up linking mapping changes to outcomes • Signal degradation when tattooing is performed too early before surgery	• Tumor-targeted tracers to add oncologic specificity (for example, anti-CEA agents) • Standardized signal-to-background metrics and imaging windows • Multicenter randomized trials with oncologic endpoints such as stage migration and disease-free survival • Dual-modality approaches combining NIR with SPECT/CT and gamma probe • AI-based nodal quantification to reduce operator dependence
TUMOR LOCALIZATION				
**Intraoperative aim**	**Advantages**	**Disadvantages**	**Limitations**	**Translational perspectives**
– Identify the primary tumor and margins; validate transection; preoperative tattooing	• Early dynamic ICG patterns differentiate malignant from benign behavior in vivo • Submucosal ICG tattoo placed within 6 days achieves near-perfect detectability • Hyperspectral imaging adds oxygenation and perfusion mapping without contrast • Targeted agent SGM-101 shows promising clinical accuracy and can alter surgical strategy	• Late or static spot fluorescence, especially ex vivo, does not reliably discriminate • Limited penetration and possible mucosal background reduce contrast for some targets • Requires direct line of sight	• Prospective clinical evidence: timing-dependent ICG signal differences are reported, but linkage to hard oncologic outcomes remains limited • Early phase/preclinical evidence: targeted and activatable probes are promising but not yet widely validated in CRC surgery • Small and heterogeneous clinical series; many early phase or preclinical datasets for targeted probes • Variable dosing and timing; no standardized TBR or SBR cut-offs or cross-platform calibration • Limited evidence that fluorescence-guided margins translate to hard outcomes	• Targeted agents such as SGM-101 for CEA, panitumumab-IRDye800CW for EGFR, EMI-137 for c-Met, activatable 6QC-ICG, and PD-L1 conjugates • Dual-channel 700 and 800 nm imaging combining targeted specificity with ICG functional sensitivity • AI and computer vision for automated detection and quantification • Integrated workflows that combine fluorescence angiography, targeted imaging, and hyperspectral imaging with harmonized reporting and metrics
DETECTION OF METASTASES				
**Intraoperative aim**	**Advantages**	**Disadvantages**	**Limitations**	**Translational perspectives**
– Detect and delineate metastases; guide margins and staging	• Liver: protruding rim fluorescence correlates with R1 margins; absence aligns with R0 and provides high negative predictive value • Reveals occult lesions missed by white light and ultrasound • Peritoneum: SGM-101 can increase lesion detection and PCI accuracy within multimodal workflows • Lung: targeted anti-CEA signals show high specificity ex vivo and aid nodule confirmation	• Liver: additional fluorescent foci may be benign, lowering specificity • Subjective interpretation of rim versus background • Lung: limited optical penetration in vivo • Peritoneum: false positives are possible with anti-CEA targeting	• Prospective interventional evidence: targeted and multimodal strategies are largely single-arm feasibility/phase II studies • Observational evidence: cohorts support margin assessment and occult-lesion detection, but specificity varies and thresholds are not standardized • Predominance of pilot or single-arm, single-center evidence • Variable doses and intervals for both ICG and targeted agents • Non-uniform imaging thresholds and camera platforms • Limited long-term outcomes and heterogeneous case mix	• Dual-channel imaging that pairs targeted 700 nm with ICG 800 nm to balance sensitivity and specificity • AI-assisted quantification, including automated rim detection • Multimodal validation embedded in protocols with ultrasound, gamma probe, and pathology • Prospective multicenter trials powered for strategy conversion, margin status, and oncologic endpoints with standardized calibration and thresholds

## Data Availability

No new data were created or analyzed in this study.

## Abbreviations

AI, artificial intelligence; ML, machine learning; AL, anastomotic leak; CME, complete mesocolic excision; CRC, colorectal cancer; CEA, carcinoembryonic antigen; c-Met, hepatocyte growth factor receptor; EGFR, epidermal growth factor receptor; PD-L1, programmed death ligand 1; FGS, fluorescence-guided surgery; HSI, hyperspectral imaging; ICG, indocyanine green; ICG-FA, indocyanine green fluorescence angiography; IMA, inferior mesenteric artery; LAR, low anterior resection; LPLN, lateral pelvic lymph nodes; NIR, near-infrared; NIRF, near-infrared fluorescence; PCI, peritoneal cancer index; SBR, signal-to-background ratio; SLN, sentinel lymph node; SPECT/CT, single photon emission computed tomography/computed tomography; TBR, tumor-to-background ratio; TME, total mesorectal excision.
